# Perinatal Psychoactive Substances Use: A Rising Perinatal Mental Health Concern

**DOI:** 10.3390/jcm12062175

**Published:** 2023-03-10

**Authors:** Gihan ELNahas, Florence Thibaut

**Affiliations:** 1 Neuropsychiatry Department, Ain Shams Medical School, Cairo 11591, Egypt; 2 University Hospital Cochin (Site Tarnier), University of Paris Cité, AP-HP, INSERM U1266, Institute of Psychiatry and Neurosciences, 75006 Paris, France

**Keywords:** women, pregnancy, perinatal mental health, alcohol, tobacco, substance use, psychoactive substance use

## Abstract

Introduction: A significant increase in psychoactive drugs use was observed in women of childbearing age and during the perinatal period worldwide. Yet, the use of illicit drugs, alcohol and tobacco during pregnancy is a serious health risk for the mother, developing fetus and newborn. Methods: This review of current trends and consequences of psychoactive substance use in the general population and in pregnant women was conducted using the English and French literature published during the years 2000 to 2022, supplemented by guidelines, meta-analyses and reviews. Results: According to current rates of prenatal substances use, it was calculated that 380,000 offspring were exposed to illicit substances, more than 500,000 to alcohol and over one million to tobacco during uterine life. Alarmingly, drug-related pregnancy-associated mortality has shown a staggering 190% rise between 2010 and 2019 in the USA. Different drugs of abuse, when used during pregnancy, increase the risk of stillbirth, neonatal abstinence syndrome and sudden infant death. Adverse effects on pregnancy include premature rupture of membranes, placental abruption, preterm birth, low birth space? weight and small-for-gestational-age infants. There is also an increased risk of morbidity and mortality for the pregnant women. Long-term negative adverse effects of perinatal exposure to substances also include a number of neurocognitive, behavioral and emotional dysfunctions in infants. Each type of substance has its own specificities, which will be briefly summarized. Conclusion: All childbearing age women must be informed about the potential harm of the prenatal use of psychoactive substances and should be encouraged to stop their use when pregnancy is planned and, at least, when pregnancy is known. Questioning women about their alcohol consumption should be systematic at the first prenatal visit and then at every prenatal visit until delivery. Multidisciplinary prevention approaches as well as intervention measures targeted to each type of psychoactive substance can save mothers’ lives and mitigate serious adversities to the offspring.

## 1. Introduction

For years, drinking, smoking and illicit drugs use have been associated with male gender, unhealthy lifestyles and risky behaviors. However, over the last few decades, the rates of psychoactive substance use and addiction have witnessed a significant rise among both men and women [1]. For example, Western adolescent girls today are 15 times more likely to begin using illicit drugs by the age of 15 as compared with their mothers [2]. Consequently, a significant increase in psychoactive drugs use was observed in women of childbearing age and during the perinatal period. Yet, the use of psychoactive substances during pregnancy is a serious health risk for the mother, developing fetus and newborn infant [3].

There is no safe use of tobacco, alcohol or illicit substances during pregnancy; even with caffeine use, the risk for infant growth restriction remains unclear, and the maximum recommended dose of caffeine during pregnancy is 200 mg/day [4].

Overall, female addictive patterns and behaviors are quite different from men. Female drinkers, for example, differ in their use of alcohol compared with their male counterparts; women tend to get drunk faster, become addicted sooner, and develop related-somatic diseases earlier than men. Among women, alcohol is the third risk factor for somatic disease and death in Europe; tobacco being the second one [5].

Males and females have different pharmacokinetics and pharmacodynamics [6]. Thus, alcohol blood concentrations are higher in women compared with men with the same amount of alcohol. Therefore, women are at a higher risk of adverse effects with lower doses of alcohol compared with men. Nicotine metabolism is faster in women (especially when combined with oral contraceptives); as a consequence, women may smoke more tobacco [7].

Moreover, hormones, vulnerability and environmental triggers differ according to sex and gender and may play an important role in differences between men and women [8]. Thus, men and women have different propensities for relapse and are susceptible to different risk factors for relapse to drug taking [9]. For example, women are less sensitive to the reinforcing effects of nicotine, in contrast, they are more sensitive to social cues. Similarly, sex/gender differences in neural correlates of cue-induced craving were reported in women and men with cocaine use disorder, with cortical–striatal–limbic hyperactivity being linked to stress cues in women but to drug cues in men [10].

Moreover, in women, heavy drinking increases the risk of injuries and accidental death, as well as practicing unsafe sex or being a victim of sexual violence [11]. Sexually transmitted diseases have more frequent and severe long-term consequences in women using alcohol and/or illicit substances. The incidence of HIV/AIDS is increasing in heterosexual women. Finally, almost 70% of women in addiction treatment, compared with 12% of men, were sexually abused as children [12].

After a short review of the current trends of psychoactive substance use in the general population, we will focus on perinatal psychoactive substance use, as a rising perinatal mental health concern.

## 2. Methods

The review was conducted using the English and French literature published during the years 2000 to 2022, supplemented by guidelines, meta-analyses and reviews. The following keywords were used: women, pregnancy, perinatal mental health, alcohol, tobacco, substance use, psychoactive substance use.

## 3. Results and Discussion

The main results were summarized, and we focused on perinatal psychoactive substances including alcohol, tobacco and illicit drug use during pregnancy.

### 3.1. Trends and Rates in the General Population

According to the World Health Organization (WHO), at the international level, European women had the highest prevalence of alcohol use disorders (harmful use and dependence) (2.9% vs. 12.6% in men) and Arabic countries the lowest (0.2% vs. 0.6% in men). Internationally, European and American adolescent women had the highest prevalence of heavy episodic drinking (22% vs. 40% of men; 7.1% vs. 29.3% of men, respectively); the Western Pacific Region (WPR) also had a high prevalence (6.1% of women compared with 18.3% of men) [13]. However, the prevalence remains globally lower than in men.

Similarly, European and American women had the highest prevalence of smoking (any tobacco product) (17.7% and 11.3% of women, respectively) compared with a worldwide mean prevalence of 7.8%. In 2012, the prevalence of cigarette smoking was close among boys and girls in Europe. In the Americas, the prevalence was even higher in girls than boys. The prevalence of smoking was close among boys and girls in Africa and the WPR when tobacco smoking other than cigarettes was considered [14].

Based on data published in 2019 by the Global Burden of Disease Collaborative Network, it was estimated that 2.4% of men were dependent on illicit drugs compared with 1.2% of women [15]. According to the latest data provided by the European Drug Report issued in 2022 to shed light on the situation in Europe, approximately 29% of adults aged 15–65 had ever used illicit drugs. The majority were men, with a prevalence of 50.5 million (60%) compared to 33.0 million of women (40%) [16].

Women had higher risks of alcohol dependence, liver cirrhosis and tissue damage compared with men [11]. The global alcohol-attributable burden of disease (in thousands of disability-adjusted life years (DALYs)) was higher in women than men when cancer (10.2 vs. 8.2); cardiovascular diseases and diabetes (33.6 vs. 10.6); and gastrointestinal diseases (17.6 vs. 12.5) were considered [17]. Moreover, the risk of breast cancer was increased when the daily dose of alcohol increased (relative risk (RR): from 5–9 with one standard drink/day to 41 with three–six standard drinks/day) [18]. There were no cardio-vascular adverse effects when the daily dose of alcohol use was less that 100 g in men compared with only 30 g in women [19]. Similarly, concerning liver cirrhosis, the RR increased by 13 when women were drinking four standard drinks of alcohol per day compared with six in men [19]. Finally, when women and men were drinking the same amount of alcohol (120 g/day), the RR of mortality due to hemorrhagic stroke was 7 and 2, respectively [20].

The gender gap in smoking has recently witnessed a notable narrowing, women are less motivated to quit and more liable to relapse if they had quit quitted? compared with men. It thus appears that the difference in the rates of health adversities due to smoking, including cardiopulmonary and lung cancer tends to minimize between both genders. In the UK, a RR of lung cancer of 21 was reported in 1.2 million of smoking women (duration of follow up of 12 years) [21]. RRs of death were 12 for respiratory diseases, 7 for lung cancer, 5 for cerebrovascular stroke and 3 for cardiovascular diseases in smoking women [22]. It is worth mentioning that some smoking-related adversities—such as blood coagulopathies and cardiac and cerebrovascular strokes—are more specific to women who use oral contraceptives.

The total burden (DALYs) related to illicit drug dependence was half in women compared with men [23]; the highest DALYs were observed with opioids in women.

### 3.2. Trends and Rates in Pregnant Women

Several studies revealed that among pregnant women, estimated past-month users of cannabis were 7%, tobacco users 10% and regular alcohol use and/or binge drinking patterns were reported in 5% [24,25]. In 2012, the results of a national survey conducted in the United States revealed that 6% of pregnant women were using illicit drugs, 16% were using tobacco and 8.5% were using alcohol. With such rates of prenatal substance use, it was calculated that 380,000 offspring were exposed to illicit substances, more than 500,000 to alcohol and over one million to tobacco during uterine life [26].

The number of women using alcohol during pregnancy varies across different countries, whereas it is estimated to be 8.7% in the US [27], it reaches 20% in countries such as France and Germany [28,29]. Using biological markers in meconium, the prevalence of alcohol use was estimated at 2.9% during the third trimester of 724 pregnant women [30]. The highest prevalence of using any amount of alcohol during pregnancy was observed in Europe (25.2%), as compared to a mean worldwide prevalence of 9.8%; the lowest prevalence in the Eastern Mediterranean Region countries (0.2%) [31]. According to Skagerström et al., 2011 [29], prepregnancy alcohol consumption, past history of sexual abuse and/or exposure to violence were the most consistent risk factors, whereas unemployment, marital status and/or education level were less consistent. Thibaut et al. [32] have also reported personal and family psychiatry history as well as relationship conflicts as risk factors.

Worldwide, approximately 20–30% of pregnant women smoke during pregnancy [33]. Thirty-five percent of 8344 pregnant women reported tobacco smoking before pregnancy and 26% maintained smoking during pregnancy, including 11% who were smoking at least 10 cigarettes per day (anonymous survey conducted in 15 European countries) [34]. According to Oh et al., 2017 [27], 15% of North American women have reported tobacco use during pregnancy. In a French cohort of 724 pregnant women, cotinine was positive in 20% of meconium at birth, whereas 17.1% and 7.8% of women reported tobacco smoking or passive exposure during the third trimester of pregnancy, respectively [30]. A study conducted in a large cohort of pregnant women reported that mothers at risk of using tobacco during pregnancy were those who used caffeine during current pregnancy, tobacco in pre-pregnancy time; those with a previous history of psychiatric or emotional disorders, a previous hospitalization for medical disorders; and mostly mothers who were lonely or whose partners were current smokers. In contrast, higher education levels and wanted pregnancies decreased the risk for tobacco smoking during pregnancy [35]. 

**Figure 1:**
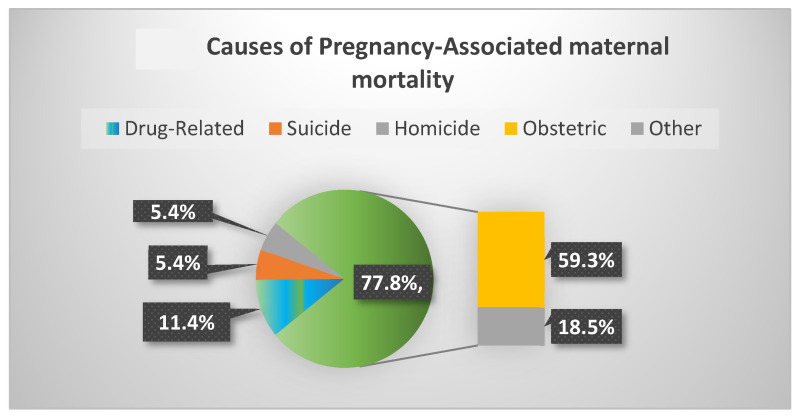
Drug-related deaths, suicide and homicide accounted for 22.2% of pregnancy-associated deaths; 11.4% of pregnancy-associated mortality was caused by drug use [36].

Finally, among 11,782 pregnancy-associated deaths identified in the United States between the years 2010 and 2019, 54.6% died from maternal or obstetric causes and 11.4% of deaths were related to drugs, 20% of which occurred during the first 6 weeks postpartum (Figure 1) [36]. Notably, three main causes accounted for non-obstetrical pregnancy-associated mortality, namely deaths related to drugs, suicide and homicide accounted for 22.2% of pregnancy-associated deaths. These latter causes of death increased during the study period. Moreover, drug-related pregnancy-associated mortality showed a staggering 190% rise over these 9 years [36].

### 3.3. Perinatal Adversities Associated with Psychoactive Substance Use in the Perinatal Period

The use and abuse of psychoactive drugs by pregnant women, including alcohol, tobacco, illicit drugs and prescription medications, has detrimental health consequences to the offspring. Different drugs of abuse when used during pregnancy could triple the risk of stillbirth. Sudden infant death (SID) is also increased, particularly for mothers who drink and smoke during the second and third trimesters of pregnancy, which raises the risk by twelvefold. In addition, neonatal abstinence syndrome (NAS) was reported in newborns whose mothers have been using opioids, prescription pain killers, tobacco, alcohol, benzodiazepines, barbiturates and even caffeine during pregnancy [37].

The adverse effects of psychoactive drugs on pregnancy include the premature rupture of membranes, placental abruption, preterm birth, low birth weight and small-for-gestational-age infants.

Moreover, the long-term negative adverse effects of perinatal exposure to psychoactive substances includes a number of neurocognitive, behavioral and emotional dysfunctions that are even aggravated by the disrupted maternal–child bonding and relationship [37,38].

It is also worth noting that substance misuse is an important factor in the morbidity and mortality of pregnant women, including death by suicide [39]. Substance use was also identified as a risk factor in the deaths of women in the first year after pregnancy [40]. 

Perinatal adversities that are encountered as a result of prenatal substance use cannot be exclusively linked to one particular substance. This is because of the frequent polysubstance use, comorbid psychiatric and medical illness, engagement in risky behaviors and the unhealthy lifestyles of those women. All these factors act as considerable confounders, making it difficult to make such solid links. 

Each type of substance has its own specificities, which will be briefly summarized below.

#### 3.3.1. Marijuana Use

Women regularly using marijuana during pregnancy are characteristically at increased risk of having other mental disorders, such as depression, panic attacks and/or anxiety disorders [37]. The use of marijuana during pregnancy has doubled over a period of 15 years (2002–2017) according to a national study conducted in North America. Marijuana use was reported more often during the first trimester than later in the course of pregnancy [41]. Studies failed to reach conclusive evidence linking marijuana use during pregnancy with miscarriage or preterm birth, but there is a strong association linking prenatal use with low birth weight. However, the role of tobacco is difficult to disentangle. This detrimental effect on fetal growth is more pronounced among frequent users, especially in the first and second trimesters [42]. There is also evidence linking marijuana use with an adverse impact on the developing fetal brain when used during pregnancy, as well as neurocognitive effects on infants and a long-lasting effect on children whose nursing mothers were using marijuana [37,43].

#### 3.3.2. Stimulants (Cocaine and Methamphetamine) Use

Women using stimulants are more likely to suffer a comorbid depressive disorder [37]. Methamphetamine use during pregnancy poses a greater risk of developing spontaneous miscarriage, preeclampsia, premature separation of the placenta and premature delivery [44]. The offspring of mothers who have used stimulants during pregnancy may have low birth weight and show increased neurobehavioral problems in childhood [45,46].

#### 3.3.3. Alcohol Use

Despite an international consensus recommending total alcohol abstinence during pregnancy, it remains an important public health issue. Prenatal alcohol exposure is the most important risk factor to avoidable neurodevelopmental disorders. Alcohol use during pregnancy was associated with numerous adverse effects (i.e., spontaneous abortion, stillbirth, weight and growth deficiencies, birth defects, prematurity and fetal alcohol spectrum disorder (FASD). FASD is characterized by growth deficiencies, craniofacial dysmorphologies and brain damage [47]. Prenatal alcohol exposure was associated with intellectual or motor disability; learning, attention and/or language disorders; poor impulse control; and hyperactivity. Fetal alcohol syndrome (FAS) is the most complete form of FASD. Fetal alcohol exposure may also be associated with later mental health diseases such as depression, anxiety, drug and alcohol use disorders, as well as behavioral disorders such as inappropriate sexual behaviors that may result in an increased rate of delinquency. Early diagnosis and management of FASD might help reduce or even prevent these adverse events [48]. Yet, FASD is largely underdiagnosed. Its diagnosis is often made late after birth (sometimes at adult age), when brain damage becomes irreversible and permanent. The worldwide prevalence of FAS is 14.6 per 10,000 people. The highest prevalence was reported in Europe (37.4 per 10,000) and in South Africa (585.3 per 10,000), the lowest was in the Arab countries (0.2–0.9 per 10,000) [31]. In Australia, the USA, Canada, South Korea, Croatia, Italy and France, according to available data one in every 67 mothers who have regularly used alcohol during pregnancy delivered a child with fetal alcohol syndrome [31]. Moderate to heavy levels of alcohol use during pregnancy (30–40 g per occasion and/or 70 g or more per week) were associated with toxic effects, but scientific evidence is still missing when lower doses were used [49]. However, all authors agreed that there is no safe amount of alcohol use during pregnancy. As a consequence, abstinence is recommended worldwide. 

#### 3.3.4. Nicotine Use

In spite of the unanimously stated adverse consequences of maternal smoking on the pregnancy itself, the neonate and the lifelong neurodevelopmental, emotional and respiratory effects (particularly asthma) on the offspring, smoking among pregnant women is not uncommon. Smoking during pregnancy is associated with preterm birth, placental hematoma, low birth weight and birth defects [50]. There is also an increased risk of SID. Neonatal nicotine abstinence syndrome including irritability, high tone and tremors may be confounded with opioid withdrawal in women who also use illicit substances. Quitting tobacco smoking at any time during pregnancy decreases the risk of adverse effects. Smoking partners may also expose the fetus and the newborn to passive smoking and their negative consequences.

#### 3.3.5. Opioid Use

Opioids have the ability to cross placental and blood–brain barriers. Obstetric complications of prenatal opioid exposure include spontaneous abortion, premature rupture of membranes, preeclampsia, abruption placentae and fetal death. Adverse neonatal outcomes include preterm birth, lower birth weight or height, reduced head circumference and SID. NAS is another frequent adverse outcome commonly observed in newborns exposed to opioids during pregnancy. In the United States, between the years 2000 and 2012 the incidence of NAS was multiplied by five. Significant impairments in cognitive, psychomotor and behavioral outcomes were reported in infants and preschool-aged children exposed prenatally. Finally, the Centers for Disease Control and Prevention draw attention to congenital malformations after opioid use during early pregnancy with a two-fold increased risk for congenital heart defects, neural tube defects and gastroschisis; for review see [51]. 

### 3.4. Management of Women with Perinatal Substance Use Problems

Pregnancy presents a unique opportunity to help women reduce and ideally stop alcohol and other psychoactive substance use during pregnancy. Healthcare providers—ideally a multidisciplinary team—should take the opportunity of probing and discussing substance misuse issues with pregnant women and not wait for their patients to bring the matter to their attention. Most pregnant women are reluctant to disclose their addiction (especially alcohol or illicit substances use) with their healthcare provider for fear of stigma, breach of confidentiality, disclosure to spouse/family and for concerns related to legal issues and their rights for childcare and guardianship. Kiel et al. [52] reported that pregnant women were unwilling to reveal cannabis use problems or even to get more information about the matter through healthcare providers. 

The perinatal healthcare team should engage in discussion with women about substance use during preconception, pregnancy and postpartum. This essentially includes an enquiry about any form of substance use early in pregnancy and at every prenatal visit. In this respect, [53] provides a number of recommendations for the identification and management of psychoactive substance misuse in pregnancy. Fathers or living partners should be included as much as possible in the withdrawal of tobacco, alcohol and/or illicit drugs use.

#### 3.4.1. Alcohol Use

The International Association for Women’s Mental Health in collaboration with the World Federation of Societies of Biological Psychiatry published guidelines for the management of alcohol use in pregnant women including a review on the risk/benefit ratio of pharmacological treatment and their indications in the prevention of withdrawal symptoms [8]. All perinatal caregivers should be aware of the risk of FASD and the risks associated with pharmacological treatment for alcohol use disorders. Information and education about the potential harm of alcohol prenatal use should be delivered to all childbearing age women and their partners. Women should be encouraged to stop alcohol when pregnancy is planned or at least when pregnancy is known. Questioning women about their alcohol consumption should be systematic at the first prenatal visit and then at every prenatal visit until delivery. At birth, in the case of a previous history of miscarriage, premature delivery or previous infant with FASD or of any doubt about alcohol use during pregnancy, FASD must be systematically searched for and alcohol metabolites should be measured in meconium of neonates [8,30]. In the case of alcohol use or pharmacological treatment for the maintenance of alcohol abstinence, breastfeeding is not recommended. 

Finally, the WHO recommendations for first-line intervention regarding the identification and management of intimate partner violence might be helpful, as violence is often associated with alcohol use disorders [54].

#### 3.4.2. Tobacco Use

The maintenance of a high level of tobacco use during pregnancy in Western countries emphasizes the importance of community education and the necessity of prevention strategies focused on the risks associated with tobacco smoking during pregnancy. The US preventive services task force recommended in 2021 to provide behavioral interventions for smoking cessation in pregnant women. They concluded that the benefit/risk ratio of pharmacotherapy interventions and the use of e-cigarettes for tobacco cessation could not be assessed in pregnant women due to insufficient scientific evidence [55]. 

Mothers should avoid smoking before breastfeeding the baby; they should wait for at least 4 h [56]. Caregivers should ask mothers to avoid exposure of their baby to secondhand smoking (especially when they smoke or live with a smoking partner).

#### 3.4.3. Illicit Drug Use

The WHO published interesting and well-documented guidelines for the identification and management of illicit substance use and substance use disorders in pregnancy [57]. Caregivers who care for pregnant women who use opioids should have access to appropriate expertise if they want to taper opioids, offer substitution therapies with buprenorphine or methadone during pregnancy and organize childbirth at a specialized facility prepared to monitor, evaluate and treat NAS at birth. NAS typically occurs within 72 h after birth. It may include tremors, irritability, excessive or high-pitched crying, hyperactive reflexes, sleep problems, seizures, yawning, sneezing, increased sweating, poor sucking and vomiting.

Breastfeeding is encouraged in women who continue to misuse substances by both the WHO [57] and the UK Department of Health [53], except if risks clearly outweigh the benefits or if the woman is also abusing cocaine or high doses of benzodiazepines. Methadone or buprenorphine treatment is not a contraindication to breastfeeding and breastfeeding may even help improve symptoms of neonatal abstinence syndrome. Support for people in treatment for opioid use disorders is critical during the postpartum period during which there is a high risk of relapse and overdose events.

The number of women with opioid use disorders at delivery increased by 131% in the US between 2010 and 2017. In 2019, according to the Centers for Disease Control and Prevention, 7% of women reported use of prescription opioid pain relievers during pregnancy. Among them, one in five reported misuse [58]. Healthcare providers and patients together should carefully weigh risks and benefits before prescription of opioid pain relievers during pregnancy.

## 4. Discussion

In this review, we attempted to bring together existing knowledge about the prevalence, trajectories, trends and practice guidelines to provide an overview of this interdisciplinary area of psychoactive substance use during pregnancy and serve as a flagship. 

The trendline of substance use among women indicates that there is a staggering rise in psychoactive substance use among women and girls as young as 15 years of age. In addition to the notable gender differences in the rates, trends, patterns of use and biopsychosocial adversities associated with psychoactive substances use [32], there is also a significant difference in preponderance across regions and across different age groups that needs to be taken into account [5,16,17,26,37]. The observed differences in prevalence across different countries would help direct international entities on how to allocate resources and where they need to redirect their lens in order to improve prevention and education. National programs developed to address substance use problems are required to be gender sensitive, attending to the specific characteristics of substance use among women and girls, taking into account the key role that violence and abuse play in the vicious circle of chronic and relapsing substances use and providing adequate care to protect and support violence survivors [54]. 

There is a wide array of adversities associated with psychoactive substance use during pregnancy in addition to the social consequences of unplanned and/or unwanted pregnancies, exposure to interpersonal violence, etc. All this indicates a need for an interdisciplinary approach to address psychoactive substance use among childbearing age women in general and during the perinatal period in particular. This should necessarily include involvement of living partners and close family in prevention and intervention approaches and essentially adopt strategies that take into account those offspring who are born with a double-pronged vulnerability that is both genetic and environmental. 

Alcohol, tobacco and illicit substance use should be systematically sought at the first antenatal visit and at all prenatal visits. Since an average of 20% of women report smoking around pregnancy, it is unanimously agreed that referral of pregnant women who smoke to smoking cessation services is essential. Importantly, our literature review emphasized that we need to turn a lens to protecting pregnant women who are exposed to secondhand smoking, whose number far exceeds those who are actively smoking. Implementing tobacco control measures, essentially 100% smoke-free indoor areas for homes and workplaces should be adopted by national bodies. Ideally, smoking partners should be included in both the awareness programs as well as cessation services. In addition, we recommend that the national and global surveillance conducted by international bodies should include measuring and monitoring the number of pregnant women actively smoking as well as those exposed to secondhand smoking. At birth, FASD must be searched for (first available cause of neurodevelopmental disorder) and alcohol metabolites should be measured in the meconium of neonates if there is any doubt about alcohol exposure during pregnancy. Medical doctors should carefully weigh risks and benefits when prescribing opioids during pregnancy. In case of illicit substance use or opioid prescription during pregnancy, health care providers should organize delivery at a facility prepared to monitor, evaluate and treat NAS. Unlike treatment of substance use disorders in men, greater prudence is required when treating pregnant women using psychoactive substances. Balancing the risks and benefits of the use of pharmacotherapy during the perinatal period is a quintessential approach in order to avoid unwarranted perinatal adversities to the mother and the developing offspring [8,57].

The longer-term impact of exposure to psychoactive substances is somewhat controversial. More studies are required to fill in our knowledge gaps and provide substantial evidence on the type and magnitude of any life-long adversities on offspring, while accounting for confounders. 

The shooting trendline of drug-related pregnancy-associated mortality (190% rise between 2010 and 2019 in the USA) is a red flag. More studies are encouraged to investigate how regularly psychiatrists, obstetricians and neonatologists are enquiring about history of substance abuse among pregnant women. In a recent study conducted to investigate patterns of practice of a random sample of 600 obstetricians [52,59], enquiry about marijuana and illegal drugs were less frequent than tobacco and alcohol, and referrals to specialized services were found to be inconsistent. Obstetricians and primary healthcare physicians should particularly be the focus of programs designed to enhance medical education and improve the standards of practice and standardized provision of care [8,57]. Moreover, health workers from different disciplines providing healthcare to women and infants may not be receiving adequate training on substance use problems among pregnant women. Perinatal substance use should be integrated in mainstream studies among medical students, nurses, midwifes, etc. [59]. In order to address this rising health issue, drug treatment programs for pregnant women need to be made available and accessible to the vulnerable populations. Given that women’s fear of possible legal or civil penalties is often the roadblock to disclosing or seeking medical help for their perinatal substance use problems, integrated care that offers standardized treatment to those women without incriminating them is quintessential to ensure engagement and adherence to treatment. The literature review also highlights the importance of including childcare and family welfare into services provided to women with perinatal substances use issues.

Psychoactive substances of all types and in all forms will inevitably pass into the breastmilk. It is thus recommended that health advisors, care providers and supporters of breastfeeding need to ask postpartum women about their use of tobacco, intake of alcohol and illicit substance use. Women with substance use history who continue to use them after delivery should be made aware of the high risk imposed on their nursed infant.

## 5. Conclusions

A significant increase in psychoactive drug use is observed in women of childbearing age and during the perinatal period worldwide. Concerning psychoactive substance use during pregnancy, there is no safe use. In addition to common adverse effects on the mother, on the pregnancy itself and on the newborn, each type of psychoactive substance has its own specific adverse effects including long-term consequences on the infant.

Pregnancy is an important window of opportunity for addressing psychoactive substance use, as the majority of pregnant women are interested in giving birth to a healthy baby. Living partners should be involved as much as possible in the withdrawal of psychoactive substance use. Multidisciplinary prevention approaches, as well as intervention measures targeted to each type of psychoactive substance can save mothers’ lives and mitigate serious adversities to the offspring.

## Authors Contribution

G.E. and F.T. both contributed equally to the draft, have approved the submitted version and agree to be personally accountable for the author’s own contributions and for ensuring that questions related to the accuracy or integrity of any part of the work, even ones in which the author was not personally involved, are appropriately investigated, resolved and documented in the literature.

## Data Availability

Not applicable.
